# The Relationship between Erythrocytes and Diabetes Mellitus

**DOI:** 10.1155/2021/6656062

**Published:** 2021-02-25

**Authors:** Yaqi Wang, Peiyuan Yang, Zhaoli Yan, Zhi Liu, Qiang Ma, Zehong Zhang, Yunxia Wang, Yan Su

**Affiliations:** ^1^Department of Biochemistry and Molecular Biology, Baotou Medical College, Baotou, 014040 Inner Mongolia, China; ^2^Department of Endocrinology, Affiliated Hospital of Inner Mongolia Medical University, Hohhot, 010050 Inner Mongolia, China

## Abstract

High blood glucose level (hyperglycemia) is a leading indicator of diabetes mellitus (DM). Erythrocytes are the most abundant cells in the circulation and the first to perceive changes in plasma composition. Long-lasting hyperglycemia affects the structure and function of erythrocytes. The detection of erythrocyte-related indicators can provide a valuable reference for the prevention, diagnosis, and treatment of DM and its complications. This paper reviews the normal structure and function of erythrocytes, the changes in erythrocytes in patients with diabetes, and the role of erythrocytes in the development of diabetic complications to provide more indicators for the early prevention of DM complications and to monitor the therapeutic effect of DM.

## 1. Introduction

Diabetes mellitus (DM) describes a group of metabolic disorders characterized by hyperglycemia and defects in insulin secretion and/or insulin action. Heredity, obesity, lack of physical activity, poor diet, stress, urbanization, impaired glucose tolerance, and hypertension may increase the risk of diabetes. Chronic hyperglycemia in patients with diabetes is associated with long-term damage and dysfunction of different organs, particularly the eyes, kidneys, nerves, heart, and blood vessels [1], which eventually results in various diabetic complications. These complications not only increase the risk of morbidity and mortality [2] but also reduce the quality of life of patients. An epidemiological survey has indicated that DM is prevalent worldwide [3]; in fact, the global prevalence of diabetes in adults over 18 years of age has increased from 4.7% in 1980 to 9.3% (463 million) in 2019 and is estimated to increase to 10.2% (578 million) by 2030 and 10.9% (700 million) by 2045 [4]. Despite advances in medical technology and extensive research on diabetes, it remains a metabolic disease that persists throughout the life and is difficult to cure. Therefore, reducing the occurrence of complications, controlling the development of complications, and improving the quality of life have become the focus of diabetes diagnosis and treatment [5]. Erythrocytes, also called red blood cells (RBCs), are the most glucose-consuming cells. In the presence of long-lasting hyperglycemia, the morphology, metabolism, and function of erythrocytes are inevitably subject to a series of changes that further affect hemorheology and microcirculation [6, 7]. Which changes occur in erythrocytes, and what is the relationship between these changes and the progression of diabetes? What significance do these changes have on the diagnosis, treatment, and prognosis of diabetes? This review aims to elaborate on the changes observed in erythrocyte in diabetic patients, the importance of erythrocytes in the development of diabetes complications, and the application of erythrocyte-related indicators to monitor disease progression and prevent complications.

## 2. Structure, Function, and Renewal of Erythrocytes

Erythrocytes are the most abundant cells in the blood. Their flexibility allows them to pass through capillaries freely, transporting oxygen to tissues and delivering carbon dioxide to the lungs. Hemoglobin (Hb), the main oxygen-carrying protein, is the most abundant protein in erythrocytes. The membrane in erythrocytes plays an important role in maintaining the stability of cell morphology and function [8]. Deformation, aggregation, and adhesion allow erythrocytes to carry oxygen. The atypical biconcave shape and small volume of erythrocytes make for a large surface area–to-volume ratio, allowing oxygen and carbon dioxide to penetrate in and out of the cell quickly and resulting in strong deformability. In addition to carrying oxygen and carbon dioxide, erythrocytes also have immune functions, such as enhancing phagocytosis, defending against infection, increasing immune adhesion, recognizing and carrying antigens, and clearing circulating immune complexes [9, 10]. Erythrocytes are produced in the red bone marrow and are released into the bloodstream after approximately 7 days of maturation. Erythrocyte production in the marrow occurs at a staggering rate of more than 2 million cells per second and is controlled by erythropoietin (EPO). The average life span of these cells is 100–120 days, and aging erythrocytes are mainly broken down in the reticuloendothelial system of the spleen and liver. The destruction and generation of human erythrocytes help maintain a dynamic balance and maintain a stable erythrocyte number [11, 12].

## 3. Clinical Erythrocyte Indicators

As an important component of blood circulation, erythrocyte is a sensitive indicator of the body's health status. Some erythrocyte parameter indicators, such as Hb concentration, hematocrit, and erythrocyte sedimentation rate (ESR), can be measured directly from the blood, whereas some parameters, such as mean cell volume (MCV), mean cell Hb, or mean cell Hb concentration, can only be calculated from multiple measured metrics (Table 1) [13]. These parameters reflect the state of erythrocytes from various angles and can be primarily used to assess the morphology, structure, function, and production of erythrocytes for the further diagnosis of some diseases [14, 15].

## 4. Erythrocyte Changes in Patients with Diabetes

### 4.1. Morphology

The normal morphology of erythrocytes is essential for their survival and ability to carry oxygen. Turchetti [16] compared the morphology of erythrocytes between normal individuals and patients with diabetes via light microscopy. In healthy subjects, the so called “bowl-shaped” erythrocytes, which are considered the most deformable cells, were the most abundant (55%) cells, followed by discocytes (44%), which are considered more rigid cells. Deformed cells, mainly echinocytes and knizocytes, do not exceed a mean value of 1%. Compared with healthy control, the deformed cell number has no significant difference with diabetics without vascular complications. However, in patients with vasculopathy, there was a statistically significant increase in discocytes (60%) and a decrease in bowl-shaped cells compared with controls. Spherocytes are reported in type 1 diabetics (T1DM), whereas both spherocytes and echinocytes were present in the peripheral blood smears of type 2 diabetics (T2DM). The authors have stated that spherocytosis, which is observed in both types of diabetes, appears to be associated with hyperglycemia, whereas echinocytes in T2DM may be related to an abnormal plasma lipid profile and increased concentration of lipid peroxides [17]. Babu [18] showed that with an increase in glucose concentration, the perimeter of erythrocytes increased, and the area of erythrocytes decreased with the increasing irregularity in the erythrocyte membrane. Furthermore, in another study, diabetics had more acanthocytes (surface blebbing cells), distorted forms, and “cup forms” (i.e., stomatocytes) compared with controls. After receiving effective treatment, the morphology of erythrocyte was restored to normal [19]. In conclusion, when the internal environment of the body changes, the number of normal, biconcave disc erythrocytes decreases as the number of deformed erythrocytes gradually increases, which further increases the risk of diabetic complications. By observing changes in the morphology and structure of erythrocytes in diabetics, a better understanding of the progression of diabetes can be achieved [20].

### 4.2. Mean Corpuscular Volume and RBC Volume Distribution Width

MCV refers to the average volume of an erythrocyte and is usually calculated indirectly. RDW is the coefficient of variation of MCV; higher RDW values reflect greater heterogeneity in MCV, which is usually caused by perturbations in erythrocyte maturation or degradation. Increases in MCV and RDW indicate inconsistency in the erythrocyte size. In clinical practice, these measurements are often combined for the differential diagnosis of anemia [21]. Blaslov et al. showed that both MCV and RDW were positively related with HbA1c and the occurrence of diabetic retinopathy [22]. MCV is also reported to be a potential risk factor of peripheral artery disease and is related to disease severity; therefore, it can be used as a predictor of diabetic macrovascular complications [23]. Higher RDW values are associated with an increased risk of cardiovascular disease and kidney disease in adults with diabetes [24]. Wang [25] noted in an experiment that RDW is associated with acute kidney injury and could be used as an independent indicator of acute kidney injury. Patients with abnormal blood glucose and kidney disease may also have abnormal RDW, and the severity of kidney disease positively correlates with RDW. Zhang [26] found that in patients with diabetic nephropathy (DN), the higher the RDW value, the more serious the glomerular injury. RDW is also related to the occurrence of urinary protein and glomerular filtration value. Increased RDW increases the risk of end-stage kidney disease in patients with DN, and RDW can be used as a predictive indicator of end-stage kidney disease for patients with DN. The erythrocytes with increased RDW usually have decreased deformability and antioxidant levels, thereby affecting the blood flow in microcirculation and increasing the inflammatory response. Usually, RDW increases when erythrocyte production is ineffective (owing to iron deficiency, chronic anemia, and vitamin B_12_ or folic acid deficiency) and decreases when erythrocytes destruction (such as hemolysis) occurs [27]. The combined use of MCV and RDW is more effective for assessing the risk of DN and should be used as an additional parameter in the risk stratification model of patients with DN. Simultaneously, both MCV and RDW can be used to predict the mortality of diabetic patients; however, the combined use of RDW and MCV can improve the predicting effect and help to provide health care services for the high risk patients. The testing results of MCV and RDW are available in the RBC count report generated by a hematology analyzer. Therefore, as economical, convenient, and minimally invasive testing methods, MCV and RDW can be widely applied for the diagnosis of diabetes [23].

### 4.3. Hemorheology

**Figure 1:**
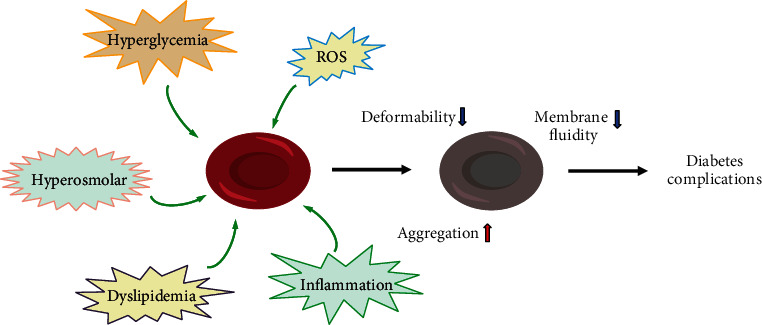
Risk of erythrocytes in diabetic patients and its effects on cellular functions. The erythrocytes in diabetic patients face multiple risks, such as hyperglycemia, hyperosmolarity, oxidative stress, inflammation, and lipid metabolism disorder, which lead to increased aggregation, reduced cell deformability, and reduced membrane fluidity. These changes in erythrocytes eventually give rise to microcirculation disorder and diabetic complications.

Hemorheology primarily focuses on the macroscopic and microscopic rheological characteristics of blood and blood vessels by observing rheological properties, such as blood viscosity, blood flow, agglutination, deformation and aggregation of erythrocytes, and aggregation and release of platelets. Among these properties, the deformability, aggregation, and fluidity of erythrocytes have been reported to have relationships with diabetes and diabetic complications (Figure 1) [28].

#### 4.3.1. Deformability

Deformability is an inherent property of erythrocytes and affects apparent blood viscosity. The deformability of erythrocytes is due to their special dynamic cell membrane shape and allows them to deliver oxygen to the tissues and organs via microcirculation to ensure effective perfusion. Glucose oxidation and protein glycation, caused by diabetes-associated hyperglycemia, can induce several modifications in the mechanical and rheological properties of erythrocytes. Lee [29] found that high glucose concentrations resulted in the glycosylation of erythrocyte membranes, thereby stiffening the glycosylated cell membranes and reducing the deformability of erythrocytes. Loyola et al. [30] observed the deformability of erythrocytes via atomic force microscopy. Their results indicated that the deformability of erythrocytes in patients with DM was reduced, and their subsequent rigidity made them difficult to pass through the microvessels and led to microcirculation disturbance. Babu et al. [31] reported that the deformability of erythrocytes was decreased in diabetic patients with lipid metabolism disorder, who are more prone to microvascular complications. Shin [32] showed that reductions in the deformability of erythrocytes also reduced their life span, and the aggregation of broken erythrocytes in microvessels impeded blood flow, which eventually led to hypoxia in body tissues. Together, these factors increase the rate of diabetic complications. Therefore, improving the deformability of erythrocytes may help prevent diabetic complications.

#### 4.3.2. Aggregation

Aggregation refers to the ability of erythrocytes to stick together. The total protein content (glycoproteins in particular) of the erythrocyte membrane is shown to decrease in patients with diabetes, whereas sialidase activity increases, which in turn, decreases sialic acids on erythrocyte surfaces. As a result, the superficial negative charge of the cells decreases, and erythrocyte aggregation increases [33]. Enhanced aggregation and adhesion also make it difficult for the assembled erythrocytes to disperse into single cells when the blood is flowing at a high shear rate. The assembled erythrocytes will block the blood vessels and cause insufficient tissue perfusion and local tissue ischemia and hypoxia, which seriously affects blood flow and oxygen transport. Critical shear stress (CSS) is a key parameter reflecting the aggregation ability of erythrocytes. Chung [34] found that in patients with DN, CSS and the aggregation capacity of erythrocytes were increased; however, blood flow velocity was decreased; conversely, patients with higher CSS had a higher risk of developing DN. Therefore, CSS can also be used as a new indicator to explain the hemorheological changes in patients with diabetic microvascular disease. Sheremet [35] showed that the erythrocyte aggregation rate in the autologous plasma and serum samples of patients with diabetic foot was significantly higher than that of normal individuals. Furthermore, the microscopic image of the erythrocyte aggregation rate in the diabetic foot demonstrated the formation of globular (pathological) aggregates in the plasma and serum. Therefore, the aggregation state of erythrocytes in the plasma and serum could be used as a risk factor of diabetic foot.

#### 4.3.3. Fluidity

Erythrocyte membrane fluidity refers to the relative lateral fluidity of proteins and lipids in the membrane structure. Many important biomembrane functions are closely related to membrane fluidity, including cellular metabolism and signal transduction, which are essential for cells to maintain their normal activities [36]. Membrane fluidity is a quantitative measurement of the lipid packing order within the membranes. Uncontrolled blood glucose fluctuations and oxidative stress (OS) are common in patients with T1DM and can damage blood cells, particularly affecting the membrane fluidity of erythrocytes, and causing diabetes complications [37, 38]. Sailaja et al. [39] reported that the molecular architecture of the erythrocyte membrane lipid bilayer was altered in patients with DM. Cordelli's study [40] found that erythrocyte liquidity could be used as an auxiliary indicator to analyze the progression of diabetes. Bianchetti et al. [41] reported that the reduced erythrocyte membrane fluidity caused by increasing nonenzymatic glycosylation, reactive oxygen species, and lipid peroxidation may be an indicator of retinopathy of T1DM. The fluidity change of diabetic erythrocytes increases their aggregation and weakens their deformability and further leads to their metabolic disorders. Therefore, increased aggregation and decreased erythrocyte deformability and fluidity caused by hyperglycemia can lead to high blood viscosity and coagulation, which result in microcirculation disorders and become an important cause of diabetic macrovascular and microvascular complications.

### 4.4. Hemoglobin

#### 4.4.1. Glycosylated Hemoglobin

Glycosylated hemoglobin (HbA1c) is one of the nonenzymatic glycosylation productions of Hb and shows the average concentration of blood glucose over the past 2–3 months. Clinically, HbA1c is usually used as an important diagnostic indicator of diabetes [42].When glucose concentration increases in the blood, it binds to the Hb in erythrocytes. Once HbA1c is formed, it does not easily decompose. HbA1c has an enhanced affinity toward O_2_; therefore, higher HbA1c concentrations lead to difficulties in releasing oxygen to cells and reduced oxygen-transporting function of erythrocytes [43]. Blaslov [22] reported a positive correlation between HbA1c and diabetic retinopathy (DR), which was caused by the reduced affinity of erythrocytes to oxygen. Local hypoxia also results in the thickening of glomerular basement membrane and thus induces DN. Philip [44] found that compared with patients without diabetes, the risk of periodontitis is increased by 2–3 times in patients with diabetes, and controlling blood glucose concentration is the key to determine this risk. In patients with T2DM, periodontitis is associated with higher HbA1c concentrations and more severe diabetic complications. Many studies have shown that HbA1c concentrations are related to both macrovascular and microvascular diseases [45]. Therefore, HbA1c is an effective indicator for preventing and monitoring diabetic complication. When combined with other erythrocyte indicators such as membrane fluidity, it can monitor DR better [41].

#### 4.4.2. Fetal Hb

Fetal Hb (HbF) is the major Hb component during intrauterine life. It rapidly decreases after birth to reach a concentration of less than 0.5% in normal children and adults. Compared with HbA, HbF has a weaker affinity for 2,3-bisphosphoglycerate, which enables the prenatal transfer of oxygen from the mother to the fetus. However, its stronger binding to oxygen makes it difficult for oxyhemoglobin F to dissociate from O_2_ and requires a much lower oxygen partial pressure, which diminishes oxygen exchange between the vasculature and tissue throughout the body. Generally, HbF is increased in some hemoglobinopathies, hypoplastic anemia, pernicious anemia, and leukemia [46]. However, HbF has been reported to increase in DM erythrocytes [47]. To compensate for the above changes, HbF binds to oxygen with a greater affinity and releases oxygen at a much lower partial pressure. It can compensatively ameliorate the hypoxic state of the body when the oxygen-carrying capacity of HbA1c decreases in patients with diabetes [48].

### 4.5. Erythrocyte Count

Erythrocyte count is the number of erythrocytes per microliter of blood and can be used to monitor the treatment of blood disorders or medications that affect erythrocytes. A low erythrocyte count indicates a decrease in oxygen-carrying cells in the blood, otherwise known as anemia; vice versa, in some high erythrocyte count cases, this may indicate that the body is compensating for some condition that is depriving the body of oxygen. In other cases, the cause may be related to diseases or drugs that alter erythrocyte production. Erythropoiesis can be stimulated by an increase in EPO synthesis in response to tissue hypoxia (kidney tissues in particular) [49]. Qadri [50] showed that although patients with diabetes had elevated erythropoiesis, the average life span of erythrocytes was shortened by 13%. Hyperglycemia and increased osmosis and OS in patients with diabetes alter the concentration of iron and protein inside and outside the erythrocytes and then activate the eryptosis pathway [51]. In patients with diabetes, the main pathways of eryptosis include the calcium ion pathway, platelet activating factor pathway, and caspase pathway; these pathways interact with one another [51–53]. A study showed that decreased erythrocyte counts in patients with T2DM were associated with microvascular complications [54]. Kim et al. reported that in the early stage of DN, anemia caused by EPO deficiency was usually an early clinical manifestation before kidney failure, and erythrocyte count could be used as an indicator of DN-related renal tubular interstitial cell damage before significant decline in the kidney function [55].

### 4.6. Erythrocyte Sedimentation Rate

ESR refers to the sedimentation velocity of erythrocytes in the blood. It fluctuates within a narrow range in healthy people and increases in many pathological conditions. Sang [56] found that the use of (Fibrinogen·ESR)/ACR could reflect the status of disease better. ESR and C-reactive protein (CRP) were also reported to play a role in monitoring the treatment of diabetic foot osteomyelitis (DFO) [57]. Lavery [58] showed that both the ESR and the CRP level were inflammatory biomarkers for evaluating a foot infection. In diabetic patients, if the ESR is <30 mm/h, the likelihood of osteomyelitis is low; however, if the ESR is >60 mm/h and the CRP level is >7.9 mg/dL, the likelihood of osteomyelitis is high, and treatment for this should be strongly considered. Xu et al. [59] reported that the ESR could be combined with the probe-to-bone test to provide a fast and early diagnosis of diabetic foot osteomyelitis. Mottaghi et al. [60] reported that a higher body mass index was associated with an increase in inflammatory markers, including CRP levels and the ESR, in diabetic polyneuropathy patients. In these patients, therapy should target weight loss among obese patients. In patients with T2DM, Guo et al. [61] reported that the ESR was independently associated with the rate and severity of diabetic kidney disease. Therefore, ESR can also be used as an indicator to evaluate the progress of patients with diabetes.

### 4.7. Metabolism

Diabetes is associated with altered cellular metabolism; however, the relationship between altered metabolism and the development of diabetic complications remains unknown [62]. Erythrocytes are important glucose-consuming cells, and glucose transporter 1 (GLUT1) mediates insulin-independent glucose transmembrane transport based on the concentration gradient in erythrocytes. As blood glucose concentration increases, more glucose enters into erythrocytes and accelerates the glucose metabolic pathways accordingly. Due to the lack of mitochondria, glycolysis is the main source of erythrocyte energy. As the product of glycolysis, ATP is the essential energy substance for a variety of biochemical reactions in erythrocytes and maintains the normal function of erythrocytes, such as transmembrane ion and lipids exchange and erythrocyte deformation [63]. The glucose uptake rate, enzyme activity, and production and utilization of intermediate metabolites and ATP in the erythrocytes of patients with diabetic were all altered [64–69]. The enhancement of the glucose metabolism in erythrocytes of diabetic patients helps in the consumption of excess blood glucose and reduces the formation of glycosylated end-products; on the other hand, it can also increase NADPH production via the pentose phosphate pathway to reduce OS in erythrocytes [70]. However, excess glucose in erythrocytes will enter the polyol pathway, and the aldose reductase- (AR-) based activation of the polyol pathway is closely related to the occurrence of diabetic complications [71]. In the polyol pathway, glucose is reduced to sorbitol by AR and then oxidized to fructose by sorbitol dehydrogenase, resulting in the accumulation of sorbitol and fructose [72]. Gupta [73] showed that high AR activity and sorbitol concentration played important roles in the pathogenesis of autonomic neuropathy in patients with T2DM. Nitric oxide (NO) produced by erythrocytes is involved in the cell deformation in the microcirculation, and the decreased NO bioavailability in erythrocytes leads to their decreased deformability and increased adhesion, resulting in microcirculation disorders [74, 75]. These changes in various metabolites in the erythrocytes of diabetic patients are involved in the occurrence of complications.

### 4.8. Oxidative Stress

OS refers to a state in which the body's oxidative and antioxidant functions are out of balance and tend to be oxidized. Under hyperglycemic conditions, autoxidation of glucose occurs, which is considered the major mechanism for free radical formation in erythrocytes. Advanced glycation end-products (AGEs) are formed via the no-enzyme glycation of protein and lipids by excessive reducing sugars, such as glucose and fructose. AGEs are considered as predeformability oxidants, which can activate several signaling pathways to produce ROS by binding to its receptor. Meanwhile, DM is often accompanied by dyslipidemia; the decrease in the glutathione (GSH) level in patients with dyslipidemia is 30% lower than that in normal people, and the average lipid peroxidation level is doubled [51]. Hyperglycemia also reduces antioxidant capacity by decreasing antioxidant levels in tissues, such as vitamin E, GSH, catalase, and SOD [75]. There is great degree of OS in diabetic patients, and the antioxidative capacity of erythrocytes is decreased [76]. Erythrocytes are vulnerable to OS, and the oxidation of structural proteins (such as cytoskeletons and membrane proteins) and functional proteins (such as enzymes) can further affect the erythrocyte function [37]. The deformability of erythrocytes damaged by OS is greatly reduced, which makes it difficult for erythrocytes to pass through microvessels and is closely related to diabetic microvascular complications. Therefore, increasing the antioxidative ability and improving the structure and function of erythrocytes may be potentially effective ways to prevent and treat DM complications.

## 5. Conclusions

**Figure 2:**
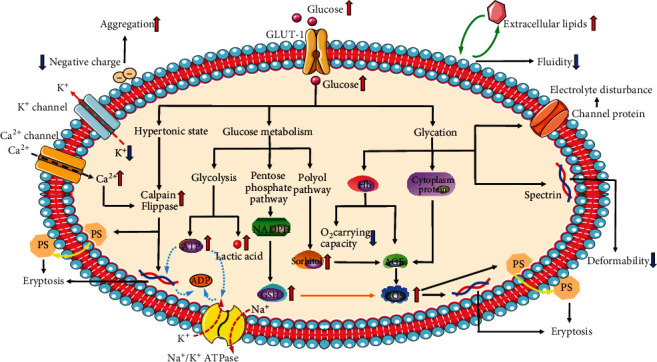
Glucose metabolism disorders in patients with diabetes profoundly affect the morphological structure and physiological functions of erythrocytes. The black arrows indicate promoting function, and the orange arrows indicate inhibitory function.

Erythrocytes are rather unique cells because they lose all organelles when mature. They only conserve a few metabolic pathways for obtaining energy and reduce the energy consumption for the key functions they need to fulfil. This makes erythrocytes highly sensitive to any disorder [77]. Glucose metabolism disorders in patients with diabetes profoundly affect the morphological structure and physiological functions of erythrocytes (Figure 2) and result in insufficient microcirculation perfusion, hypoxia, and OS, which promoting the occurrence of diabetic complications and reducing the quality of life of diabetic patients. Given the important role of erythrocytes in the pathological development of diabetic complications, the corresponding detection indicators of erythrocytes also correlated with the occurrence and progression of these complications. There have been many breakthroughs in the field of diabetes research; however, the prevention and treatment of its complications remain an important health problem. As one of the cells that can sense blood glucose changes early and continuously, erythrocyte-related indicators can provide more clinical information and can be used to monitor the progression of diabetes and its complications.

## Figures and Tables

**Table 1:** Clinical erythrocyte parameters.

Parameters	Detection method	Reference values	Clinical significance
Erythrocyte count	Automatic blood cell analyzer	Male: 4–5.5 × 10^12^/L Female: 3.5–5 × 10^12^/L Newborn: 6–7 × 10^12^/L	Reflects the number of erythrocytes in the body
Hb concentration (Hb)	Automatic blood cell analyzer	Male: 120–170 g/L Female: 110–150 g/L Newborn: 170–200 g/L	Reflects the Hb concentration of whole blood
Hematocrit (Hct)	Automatic blood cell analyzer	Male: 0.4–0.5 Female: 0.37–0.48	Refers to the percentage of erythrocytes in the whole blood
Mean corpuscular volume (MCV)	Automatic blood cell analyzer	Adult: 80–100 fl Newborn: 86–120 fl	Refers to the average volume of erythrocytes
Mean corpuscular Hb (MCH)	Automatic blood cell analyzer	Adult: 26–34 pg Newborn: 27–36 pg	Refers to the average amount of Hb in erythrocytes
Mean corpuscular Hb concentration (MCHC)	Automatic blood cell analyzer	Adult: 320–360 g/L Newborn: 250–370 g/L	Refers to the average Hb concentration in erythrocytes
RBC volume distribution width (RDW)	Automatic blood cell analyzer	<15	Refers to the heterogenicity of the size of erythrocytes
Reticulocyte count (Ret)	Automatic blood cell analyzer	Adult: (25–85) × 109/L Newborn: (85–255) × 109/L	An indicator of the hematopoietic function of the bone marrow
Erythrocyte sedimentation rate (ESR)	Westergren method/automatic ESR analyzer	Adult: <50 years old: 0–20 mm >50 years old: 0–30 mm >85 years old: 0–42 mm Child: 0–10 mm (women sink faster than men)	The rate of erythrocyte sedimentation per unit time is associated with a variety of diseases
RBC deformation index (RDI)	Blood rheometer	0.47–0.55	The deformability of erythrocytes
RBC rigidity index (RRI)	Blood rheometer	Male: 7.16 Female: 7.14	An indicator of erythrocyte membrane flexibility
RBC aggregation index (AI)	Erythrocyte electrophoretic time	1.44–3.62	An indicator of blood viscosity; often used to diagnose thrombosis
Glycosylated Hb (GHb/HbA1c)	Affinity chromatography/high performance liquid chromatography (HPLC)	4%–6%	Reflects the body's average blood sugar levels over the past 6–8 weeks
Erythrocyte morphology	Blood smear	Double concave disk, the average diameter was 7.2 *μ*m, pale pink, one-third of the center is the physiological light staining area, and there are no abnormal structures in the cytoplasm	Observe the size, morphology, and internal structure of erythrocytes
Membrane fluidity	Fluorescent Laurdan probe	Generalized polarization (GP) within the normal range	Erythrocyte membrane fluidity could provide a complementary index to monitor the development of disease
